# Advanced endometrial cancer in the gulf cooperation council: a modified-Delphi consensus on unmet needs, molecular testing, and treatment sequencing

**DOI:** 10.3389/fonc.2026.1916197

**Published:** 2026-09-24

**Authors:** Hassan Jaafar, Aref Chehal, Basel Altrabulsi, Dalia El Shorbagy, Dina Hamza, Fathi Azribi, Hind Elmalik, Maha Alsindi, Mohamed Ashour, Syed Hammad Tirmazy, Emad Dawoud

**Affiliations:** 1 Department of Oncology, Burjeel Medical City, Abu Dhabi, United Arab Emirates; 2 Department of Oncology, Sheikh Shakhbout Medical City, Abu Dhabi, United Arab Emirates; 3 Pathology and Laboratory Medicine Institute, Cleveland Clinic Abu Dhabi, Abu Dhabi, United Arab Emirates; 4 Department of Oncology, Dubai Hospital, Dubai, United Arab Emirates; 5 Department of Oncology, American Hospital, Dubai, United Arab Emirates; 6 National Center for Cancer Care and Research, Department of Oncology, Hamad Medical Corporation, Doha, Qatar; 7 Department of Oncology, Salmaniya Medical Complex, Manama, Bahrain; 8 Department of Medical Oncology, Kuwait Cancer Control Centre, Farwaniya, Kuwait; 9 Department of Oncology, Tawam Hospital, Al Ain, United Arab Emirates

**Keywords:** endometrial cancer, gulf cooperation council, immunotherapy, modified Delphi consensus, molecular testing

## Abstract

**Background:**

Advanced endometrial cancer (EC), including primary unresectable stage III–IV, metastatic, and recurrent disease, is an increasing clinical challenge in the Gulf Cooperation Council (GCC). Rising cardiometabolic risk, heterogeneous referral pathways, variable access to molecular diagnostics, and inconsistent uptake of biomarker-driven therapy contribute to variation in care. This consensus aimed to identify GCC-specific unmet needs and develop regionally adapted recommendations for the diagnosis and management of advanced or recurrent EC.

**Methods:**

A structured, modified Delphi process was conducted with 12 experts from Bahrain, Kuwait, Qatar, and the United Arab Emirates. Candidate statements were developed from a targeted PubMed literature review and evaluated through 2 rounds of anonymous voting using predefined consensus thresholds. A consensus was considered when voting reached a 75%–89% agreement, a strong consensus was defined as ≥90% agreement, and no consensus was defined as <75% agreement.

**Results:**

Consensus was achieved on 31 statements. The panel identified key regional gaps, including increasing EC burden, limited GCC-specific molecular and real-world data, inconsistent implementation of molecular testing, variable access to immunotherapy and targeted therapies, and delays in referral to specialized centers. GCC experts recommended routine molecular classification at diagnosis, standardized diagnostic work-up and staging aligned with FIGO 2023, multidisciplinary tumor board review before any clinical decision, carboplatin-paclitaxel plus immunotherapy as the preferred first-line standard for eligible patients, biomarker-informed treatment selection in the post-platinum setting, and early integration of supportive and palliative care. Additional priorities included clinician education, registry development, patient-reported outcomes, and implementation tools to improve real-world quality of care. Final recommendations were classified as international guideline-aligned, GCC-specific adaptation, or expert-opinion recommendation.

**Conclusion:**

This consensus provides a practical, GCC-adapted framework to support more consistent, biomarker-informed, and multidisciplinary management of advanced or recurrent EC across the region.

## 1 Introduction

Endometrial cancer (EC) is the sixth most common malignancy in women worldwide and is considered a significant public health burden (1). Globally, EC accounted for approximately 420,368 new cases and 97,723 deaths in 2022, and its burden is projected to increase substantially, reaching an estimate of 597,000 new cases and more than 150,000 deaths by 2040 (2, 3). Although most women present with localized disease, nearly 27% of patients present with regional disease or distant metastasis at diagnosis (4). Patients with stage III–IV EC continue to have poor long-term survival, with a median overall survival (OS) of 34.0 months (5). This highlights the unmet need for better risk stratification and treatment selection.

The management of EC has evolved substantially with the integration of molecular classification into clinical practice. Molecular subgroups, including POLE-mutated (POLEmut), mismatch repair-deficient/microsatellite instability-high (MMRd/MSI-H), p53-abnormal (p53abn) tumors, and no specific molecular profile (NSMP) tumors, have evident prognostic and therapeutic implications (6, 7). MMRd/MSI-H status has become clinically important because it identifies patients more likely to benefit from immune checkpoint inhibitor-based therapies (6, 7). HER2 overexpression/amplification also identifies EC subsets, such as serous and p53abn tumors, eligible for HER2-directed therapy. These advances have led international guidelines to recommend molecular classification at diagnosis and to include molecular classification in treatment decisions for advanced and recurrent EC (8, 9).

Recent clinical trials have changed the treatment landscape of advanced and recurrent EC, particularly through biomarker-informed use of immunotherapy and targeted agents (10). However, the therapeutic benefit is not similar across molecular subgroups. For instance, MMRd/MSI-H tumors show the greatest benefit from immune checkpoint inhibitor-based therapy, whereas the benefit in pMMR tumors is more modest and should be interpreted depending on its trial design, toxicity, patient fitness, approval status, and availability. The implementation of molecularly guided care remains inconsistent across health systems, with variable access to testing, specialist referral, and newer therapies (11). These challenges are relevant in regions where care pathways are heterogeneous and local real-world evidence is limited (12).

In the Gulf Cooperation Council (GCC) countries, EC represents an important and growing gynecologic cancer burden, with an estimate of 1,310 new cases in 2022, and a predicted increase reaching 3,310 new cases by 2040 (3). Despite the burden of EC in the region, there is a lack of region-specific recommendations to standardize molecular testing and treatment sequencing in advanced settings. This modified Delphi consensus aims to identify how evidence-based recommendations can be implemented in GCC healthcare systems, where referral structures, molecular testing capacity, reimbursement pathways, and access to immunotherapy and targeted therapies vary between countries and between public and private sectors. Accordingly, this modified Delphi consensus was conducted to define the unmet needs and contemporary management challenges of advanced or recurrent EC in the GCC and to develop evidence-based, regionally adapted recommendations to standardize molecular testing, treatment sequencing, and care pathways in routine clinical care.

## 2 Methods

### 2.1 Study design

This consensus was developed using a structured, modified Delphi process to generate regionally adapted recommendations for the management of advanced or recurrent EC in the GCC. The consensus process was designed to distinguish between recommendations that are directly aligned with international standards, recommendations requiring GCC-specific adaptation, and recommendations based on expert opinion. This aims to differentiate between expert agreement and the maturity of supporting evidence.

### 2.2 Panel recruitment

A regional expert panel was convened from four GCC countries: Bahrain, Kuwait, Qatar, and the United Arab Emirates. The panel comprised 12 experts, including 11 medical oncologists and one clinical pathologist, all of whom had at least 10 years of experience in EC management or molecular diagnostics and active academic or research involvement in the field. The panel was selected to reflect experts involved in systemic therapy and molecular decision-making for advanced EC in the GCC.

### 2.3 Literature review

A scientific steering committee conducted a targeted literature review on January 15, 2026, in MEDLINE via PubMed using a combination of Medical Subject Headings (MeSH) and free-text terms. The search focused on five predefined domains: epidemiology and unmet needs; diagnostic work-up and staging, including molecular testing; first-line systemic therapy; second-line and later-line treatment; and supportive, palliative, and implementation considerations (**Supplementary Material 1**). Evidence was screened for relevance, with priority given to randomized controlled trials, meta-analyses, and current international guidelines, supplemented by high-quality observational studies where higher-level evidence was limited.

### 2.4 Statement development

Based on this review, the steering committee drafted candidate statements, which were refined for clarity, clinical applicability, and regional relevance. Statements covered EC epidemiology and unmet needs, molecular testing and staging, first-line treatment, subsequent treatment, supportive care, and implementation priorities. Each statement was categorized during development as: (1) international guideline-aligned, (2) GCC-specific adaptation, or (3) expert-opinion recommendation. Statements were refined for clarity, clinical applicability, evidence alignment, and regional relevance.

### 2.5 Voting rounds and expert meeting

Statements were circulated to panelists for anonymous scoring through a structured electronic questionnaire. Each statement was rated on a 5-point Likert scale ranging from 1 (strongly disagree) to 5 (strongly agree), with optional free-text comments. Overall agreement was calculated as the proportion of experts selecting “agree” or “strongly agree” among valid non-neutral responses; neutral/abstain responses were excluded from the denominator. Consensus thresholds were predefined: strong consensus as ≥90% of panelists scoring 4–5, consensus as 75%–89% agreement, and no consensus as <75% agreement. Following the first voting round, the panel met on January 30, 2026, to review statements that had not reached consensus and refine their wording as needed. Revised or newly proposed statements were then submitted to a second round of anonymous voting using the same predefined thresholds. The twelve experts were included in both voting rounds and the panel meeting. The twelve voters were retained as the denominator to ensure transparency of the participating experts in the consensus process. The final consensus set was based on the combination of results from Rounds 1 and 2, and all panelists reviewed and approved the final statements stated in the manuscript (**Supplementary Table 1**).

## 3 Results

### 3.1 Current status of advanced endometrial cancer in the GCC

#### 3.1.1 Epidemiology and presentation

The burden of EC has been increasing in the GCC over the past decades, with age-standardized incidence rates between 1.3 and 5.5 per 100,000 women in 2000 (13, 14). GLOBOCAN 2022 estimated age-standardized incidence rates ranging from 3.5 to 10.3 per 100,000 women across GCC countries (3). This increasing pattern is partly driven by the rising prevalence of established EC risk factors in the region, including obesity, diabetes, and metabolic syndrome (15–18). A meta-analysis on GCC populations reported a substantial burden of metabolic syndrome, with a weighted prevalence of 27.3% (19). Additionally, the prevalence of obesity in GCC countries is among the highest regions globally, with reported rates ranging between 32% and 38% (20). The available registry and institutional data support the panel’s view that EC incidence has risen across the GCC in recent decades.

The stage distribution of EC is broadly similar to international patterns, with stage III disease accounting for approximately 16%–21.5% of cases and stage IV disease accounting for 8%–12% (21–23). Regional studies suggest that women with advanced EC in the GCC may present at a relatively younger age than typically reported in Western populations. This may be partly attributed to the younger demographic structure of GCC populations, as well as the large expatriate population residing in the region (22, 23). Data from the GCC were consistent with international cohorts, showing that endometrioid adenocarcinoma remains the predominant histologic subtype of EC, accounting for up to 73% of cases (23).

#### 3.1.2 Clinical unmet needs

The management of advanced or recurrent EC has become increasingly complex with the integration of molecular classification and biomarker-directed systemic therapy (24). In the last decade, GCC healthcare systems have made progress in adopting molecularly guided care. This was driven by the expansion in molecular pathology capabilities and the continuous enhancement in healthcare infrastructure (23). However, implementation across the GCC remains variable, and access to comprehensive molecular testing remains inconsistent (25).

The panel acknowledged the need for GCC-specific data on molecular subtype distribution and real-world outcomes. GCC-specific data could support decision-making and regional benchmarking based on generated evidence. In addition, delayed referral to specialized gynecologic oncology services and variability in awareness among non-specialist providers may contribute to suboptimal care pathways (26). Multiple studies indicated that prolonged intervals from diagnosis to treatment are associated with worse OS, higher risk of recurrence, and increased rates of adjuvant pelvic radiation (27, 28). Country-level descriptions of oncology care delivery in Oman, Kuwait, and Bahrain highlight differences in service, resourcing, and access pathways that can affect timely access to advanced diagnostics and systemic therapies (29). To address these gaps, there is a need for clearer referral pathways for suspected EC cases, earlier triage of women with high-risk symptoms, faster access to endometrial sampling and pathology review, and reduced turnaround times for diagnostic and molecular testing. The panel also highlighted the importance of increasing awareness among healthcare providers and stronger multidisciplinary coordination across gynecology, oncology, pathology, and molecular diagnostics. Collectively, these priorities support the need for GCC-adapted recommendations to improve molecular testing pathways, standardize treatment sequencing, and strengthen multidisciplinary care for advanced EC (**Table 1**).

**Table 1:** Consensus statements describing the current status of advanced endometrial cancer across GCC countries.

No	Consensus statement	Agreement^ǂ^
1	The incidence of advanced endometrial cancer in the Gulf region has increased over the past decade, primarily driven by rising obesity, diabetes, and metabolic syndrome prevalence.^*^	Strong consensus (100%)
2	The clinical stage of endometrial cancer in the Gulf aligns with global benchmarks; however, patients with advanced stages present at a younger age, due to the younger demographic of the region’s population.^*^	Consensus (80%)
3	Delays in referral from primary or secondary care to specialized gynecologic oncology centers remain a major barrier to optimal patient outcomes.^*^	Strong consensus (91.7%)
4	Limited awareness among non-specialist physicians contributes to delayed diagnosis and delayed treatment escalation in advanced endometrial cancer.^*^	Strong consensus (100%)
5	There is an unmet need for Gulf-specific epidemiological and real-world outcomes data to inform clinical decision-making in advanced endometrial cancer.^*^	Strong consensus (100%)
6	Endometrioid adenocarcinoma remains the most common histological subtype of advanced endometrial cancer in Gulf countries.^*^	Strong consensus (91.7%)
7	Comprehensive molecular testing for endometrial cancer remains suboptimal across the Gulf region, necessitating expanded coverage, improved accessibility, and standardized testing protocols.^*^	Consensus (80%)
8	Real-world data on the molecular subtypes of advanced endometrial cancer in the Gulf are limited.^*^	Strong consensus (100%)
9	Access to immunotherapy and targeted therapies for advanced endometrial cancer varies significantly between public and private healthcare setups in Gulf countries.^*^	Strong consensus (100%)

*GCC-specific adaptation.

**^ǂ^**Agreement reflects the proportion of panelists voting agree/strongly agree (scores 4–5 on a 5-point Likert scale). “Consensus” denotes 75–89% agreement, and “Strong consensus” denotes ≥90% agreement. Agreement was calculated from 12 voters, with neutral/abstain responses excluded from the denominator for the overall agreement percentage.

EC, endometrial cancer; GCC, Gulf Cooperation Council.

### 3.2 Best practices for diagnostic work-up of advanced endometrial cancer in the GCC

#### 3.2.1 Role of molecular classification

Molecular classification is now a core component of EC care, shifting from histology-led risk assessment toward integrated molecular stratification, which refines prognosis and directly informs treatment selection (7, 11). The Cancer Genome Atlas (TCGA) framework has established biologically distinct EC subgroups with different clinical outcomes, and subsequent studies have confirmed the prognostic relevance of POLEmut, MMRd, p53abn, and NSMP tumors (7). The PORTEC-3 study (n=410) showed a distribution of 23% p53abn, 12% POLEmut, 33% MMRd, and 32% NSMP (30).

Molecular work-up at diagnosis should include the core classifiers required for risk stratification and treatment planning, with additional biomarker testing guided by histology and clinical context. MMRd/MSI-H status is the most established predictive biomarker for benefit from immune checkpoint inhibition, as supported by first-line trials such as DUO-E, RUBY, and NRG-GY018, as well as recurrent-setting data from GARNET (31–34). Current international guidelines support that all patients presenting with advanced EC should undergo molecular testing at initial diagnosis (11).

In the final validation of the ProMisE classifier in a large population-based cohort (n=452), 28% of tumors were MMRd. In comparison, the remaining 72% were distributed across POLEmut, p53abn, and p53 wild-type/NSMP subgroups (35). Uterine serous carcinoma accounts for approximately 5–10% of EC diagnoses, yet it is responsible for approximately 40% of EC-related deaths (36). Therefore, advanced EC should be stratified according to histologic grade, histologic subtype, and molecular profile. High-grade endometrioid tumors, p53abn tumors, and non-endometroid histologies, including serous, clear cell, carcinosarcoma, high-grade endometrioid, and p53abn, should be recognized as aggressive subgroups requiring intensified management plans. Accordingly, molecular classification was recommended at the time of diagnosis for all patients with advanced or recurrent EC, upfront MMR testing, intensified management of high-risk/p53abn tumors, and risk-adapted assessment of heterogeneous pMMR disease.

#### 3.2.2 Diagnosis and radiological staging

Histologic confirmation remains the diagnostic cornerstone of EC, but biopsy strategy should also ensure adequate tissue for molecular testing. Pipelle biopsy, dilatation and curettage (D&C) are both acceptable diagnostic approaches. A recent meta-analysis found that both Pipelle biopsy and conventional D&C had high diagnostic accuracy for EC, with pooled sensitivity and specificity of 0.774 and 0.985 for Pipelle, and 0.880 and 0.984 for D&C, respectively (37). Hysteroscopy-guided sampling is particularly valuable when focal intrauterine lesions are suspected or prior sampling is nondiagnostic. Another meta-analysis found that hysteroscopy-guided sampling was more likely to obtain an adequate specimen and less likely to miss EC or hyperplasia, compared to blind sampling (38). Baseline imaging to define the extent and spread of disease should follow a standardized approach across GCC centers, based on the patient’s clinical presentation and suspected disease burden. Contrast-enhanced CT of the chest, abdomen, and pelvis is proposed as the standard baseline imaging modality in advanced or recurrent EC, with pelvic MRI or PET/CT considered in selected cases for additional clarification of locoregional or metastatic disease. Aligned with the ESGO–ESTRO–ESP 2025 guideline update, the GCC panel supports adopting FIGO 2023 as the most appropriate staging framework because it better aligns anatomic extent with tumor biology and supports integration of molecular classification into prognostic assessment and treatment planning (**Table 2**) (8, 11).

**Table 2:** Consensus statements describing the best practice for the diagnosis and molecular classification of advanced endometrial cancer across GCC countries.

No	Consensus statement	Agreement^ǂ^
10	Hysteroscopy-guided endometrial sampling is the first choice of biopsy method; however, sampling using Pipelle (suction curettage) and D&C (dilatation and curettage) are both acceptable approaches for the histological diagnosis of endometrial cancer.^*^	Consensus (80%)
11	Radiologic staging and baseline disease assessment should be unified across oncology centers in the Gulf region by adopting the FIGO 2023 classification and incorporating molecular subtyping as the standard staging system.^*^	Strong consensus (100%)
12	Universal molecular classification using MMR, TP53, HER2, and ER testing should be performed for all patients with advanced or recurrent endometrial cancer at diagnosis, for treatment planning and guidance.^*^	Strong consensus (100%)
13	MMR testing should be performed at initial diagnosis rather than recurrence, as identifying MMRd/MSI-H status is essential for prognostic assessment and the selection of immunotherapy-based treatments in advanced or recurrent endometrial cancer.^*^	Strong consensus (91.7%)
14	Proficient mismatch repair (pMMR) endometrial cancer should be recognized as a biologically and clinically heterogeneous group, with prognosis guided by integrated clinicopathologic and molecular risk factors rather than MMR status alone.^*^	Strong consensus (100%)
15	High-risk histological subtypes of advanced endometrial cancer, including serous, clear cell, carcinosarcoma, and p53abn tumors, should be differentiated as a distinct clinical subgroup and managed with intensified systemic treatment strategies.^*^	Strong consensus (100%)

*International guideline-aligned.

**^ǂ^**Agreement reflects the proportion of panelists voting agree/strongly agree (scores 4–5 on a 5-point Likert scale). “Consensus” denotes 75–89% agreement and “Strong consensus” denotes ≥90% agreement. Agreement was calculated from 12 voters, with neutral/abstain responses excluded from the denominator for the overall agreement percentage.

D&C, dilatation and curettage; MMRd, deficient mismatch repair; EC, endometrial cancer; ER, estrogen receptor; FIGO, International Federation of Gynecology and Obstetrics; GCC, Gulf Cooperation Council; HER2, human epidermal growth factor receptor 2 (ERBB2); LoE, level of evidence; MMR, mismatch repair; MSI-H, microsatellite instability–high; pMMR, proficient mismatch repair; TP53, tumor protein p53 gene.

### 3.3 Best practices for management of advanced endometrial cancer in the gulf

Multidisciplinary management is an essential requirement in all cases with advanced or recurrent EC, with high emphasis on patients with high-risk histology, recurrent disease, or treatment decisions requiring integration of pathology, molecular classification, imaging, surgery, radiotherapy, and systemic therapy (11). Accordingly, management plans for all newly diagnosed EC should be discussed in a specialized gynecologic oncology setting and, whenever feasible, reviewed in a multidisciplinary tumor board.

#### 3.3.1 First-line systemic treatment standards

First-line treatment in advanced or recurrent EC should be guided primarily by molecular subtype, clinical presentation, comorbidities, performance status, tumor burden, treatment goals, and treatment availability (**Figure 1**). Across phase III trials, adding an immune checkpoint inhibitor to carboplatin-paclitaxel improved PFS in advanced or recurrent EC. The greatest benefit was observed in MMRd/MSI-H tumors and a more modest but clinically relevant benefit in pMMR disease. Among phase III trials, the RUBY trial provided the highest OS benefit in the overall population, the AtTEnd trial did not demonstrate an OS benefit, and other trials were not primarily powered for OS.

**Figure 1:**
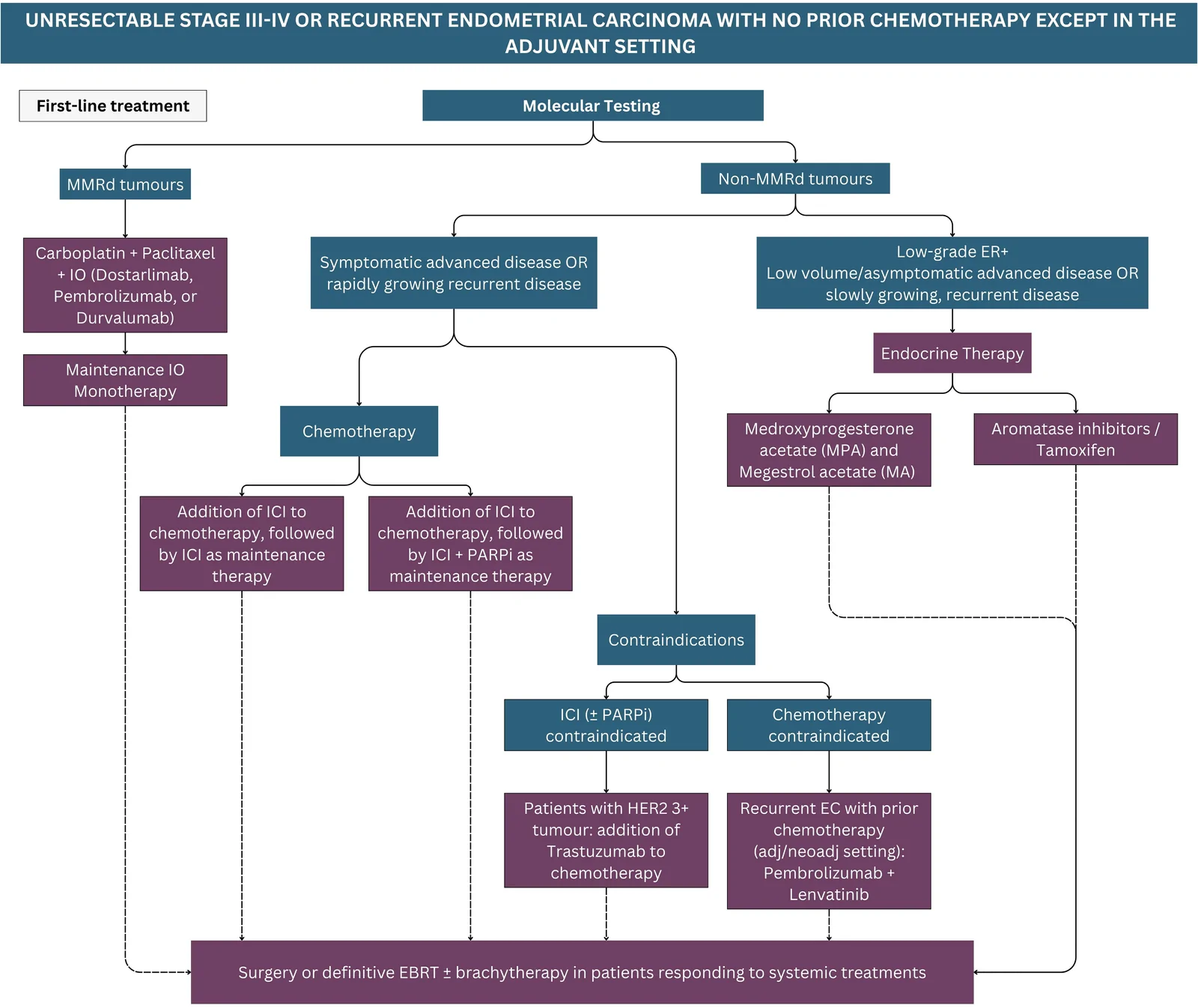
First-line treatment algorithm for unresectable stage III–IV or recurrent endometrial carcinoma based on molecular classification and clinical presentation (11) EBRT, external beam radiotherapy; EC, endometrial cancer; ER+, estrogen receptor-positive; HER2, human epidermal growth factor receptor 2; ICI, immune checkpoint inhibitor; IO, immuno-oncology; MA, megestrol acetate; MMRd, mismatch repair-deficient; MPA, medroxyprogesterone acetate; PARPi, poly(ADP-ribose) polymerase inhibitor.

In NRG-GY018, pembrolizumab improved PFS in MMRd tumors (HR 0.30; 95% CI 0.19–0.48; 12-month PFS 74% vs 38%) and also in pMMR tumors (HR 0.54; 95% CI 0.41–0.71) (32). In the RUBY trial, dostarlimab improved 24-month PFS in MMRd/MSI-H tumors (HR 0.28; 95% CI 0.16–0.50; 61.4% vs 15.7%) (33). In the AtTEnd trial, atezolizumab prolonged PFS, particularly in MMRd tumors (HR 0.36; 95% CI 0.23–0.57) (39). In the DUO-E trial, durvalumab improved PFS in both MMRd (HR 0.42; 95% CI 0.22–0.80) and pMMR disease (HR 0.77; 95% CI 0.60–0.97) (34). These trials establish chemo-immunotherapy as an evidence-based first-line strategy across MMR subgroups, while showing variable grades of benefit according to MMR status.

Therefore, carboplatin-paclitaxel plus an approved immune checkpoint inhibitor should be regarded as the preferred first-line standard for all eligible patients with advanced or first recurrent EC, with the greatest benefit in MMRd/MSI-H tumors. In pMMR disease, the benefit is more modest, and treatment should be individualized according to several factors, including benefit, toxicity, patient preference, comorbidities, and access across GCC healthcare settings.

The combination of PARP inhibitors with immunotherapy as maintenance treatment in selected high-risk pMMR tumors was recommended. PARP inhibition may enhance genomic stress and tumor immunogenicity, providing a biological rationale for combination with immune checkpoint blockade. In the DUO-E trial, carboplatin-paclitaxel plus durvalumab followed by maintenance durvalumab plus olaparib improved PFS versus chemotherapy alone in the intention-to-treat population (HR 0.55; 95% CI, 0.43–0.69), with the clinically most relevant outcomes arising from the predominantly pMMR population (34). The treatment effect in the pMMR population provided the main clinical basis for considering this approach. This was supported by the network meta-analysis conducted by Villacampa et al., where adding PARP inhibition to immunotherapy provided the most benefit in the p53abn subgroup (HR 0.47; 95% CI, 0.40–0.94) (40).

Therefore, immunotherapy plus PARP inhibitor maintenance may be considered in selected, medically fit patients with high-risk pMMR EC after first-line chemo-immunotherapy, particularly when adverse biological features such as p53abn or high-grade serous histology are present. This strategy should not be routinely applied to all patients with pMMR disease. The decision should also consider prior response to first-line treatment, performance status, toxicity, comorbidities, patient preference, regulatory approval, and local access. An MDT decision on the initiation of PARP inhibitors is recommended after discussing the supporting evidence, where each decision should be individualized.

Within pMMR disease, additional biologic refinement remains clinically necessary in the first-line setting. ER status is most useful in patients with low-volume, indolent, or asymptomatic advanced or recurrent disease, where endocrine therapy may be considered in ER-positive tumors. p53 status is considered a marker to assess prognosis, with no role in first-line treatment selection in advanced or recurrent EC (30). However, p53 status alone should not be interpreted as a definitive predictive biomarker for selecting specific first-line regimens outside the context of available evidence and local approvals.

#### 3.3.2 Second-line and subsequent treatment standards

After progression on first-line therapy in advanced or recurrent EC, treatment should continue to be biomarker-informed. Currently, there is no universally accepted standard of care after progression on prior checkpoint inhibitor-based first-line therapy. Treatment sequencing after prior immune checkpoint inhibitor exposure should be individualized according to prior response, duration of benefit, platinum-free interval, MMR/MSI status, HER2 status, ER status, p53 status, disease pace, symptom burden, performance status, patient preferences, and treatment goals.

Pembrolizumab plus lenvatinib remains an evidence-based option for patients with pMMR tumors who are immunotherapy-naïve at progression. This is supported by the 309/KEYNOTE-775, where a median PFS of 6.6 versus 3.8 months (HR 0.60; 95% CI, 0.50–0.72) and a median OS of 17.4 versus 12.0 months (HR 0.68; 95% CI, 0.56–0.84) were achieved compared with physician’s-choice chemotherapy (41). In MMRd/MSI-H tumors, checkpoint inhibitor monotherapy remains a relevant option after prior platinum exposure in immunotherapy-naïve patients, as evident in the KEYNOTE-158 and GARNET trials, and supported by other real-world studies (31, 42, 43). However, the evidence for pembrolizumab-lenvatinib or checkpoint inhibitor rechallenge after prior first-line immunotherapy is limited, and such decisions should be multidisciplinary and not routine.

Beyond these immunotherapy-naïve biomarker-defined pathways, there is no universally accepted standard of care in the second-line setting. The use of antibody-drug conjugates and targeted therapies in advanced EC is increasingly biomarker-driven, particularly in HER2-positive, serous carcinoma, carcinosarcoma, and other molecularly selected subgroups. Therefore, HER2 testing should be performed at diagnosis or recurrence in advanced or recurrent serous carcinoma, carcinosarcoma with serous or p53abn features, and selected high-grade p53abn or non-endometrioid tumors where HER2-directed therapy may be considered. In addition, repeating HER2 testing may be appropriate if prior tissue is inadequate, outdated, or discordant with current disease biology. While trastuzumab deruxtecan demonstrated high response rates in HER2-expressing disease, including an ORR of 84.6% in HER2 IHC 3+ tumors in DESTINY-PanTumor02 and 54.5% in HER2-high and 70.0% in HER2-low uterine carcinosarcoma in STATICE (44, 45). Beyond HER2, other targeted approaches remain promising but less established, with sacituzumab govitecan achieving an ORR of 22% and a median PFS of 4.8 months in heavily pretreated advanced EC, while rare alterations such as NTRK fusions may support tissue-agnostic targeted therapy when present (46). These approaches are promising and should be considered in accordance with biomarker profile, availability, and evolving evidence.

In addition, immune checkpoint inhibitor rechallenge should not be a standard strategy. It could be considered in highly selected MMRd/MSI-H cases with prior ICI benefit, such as durable prior benefit, limited progression, long treatment-free interval, preserved performance status, and limited alternatives. Platinum rechallenge should be reserved for selected cases rather than used routinely. Other options, such as weekly paclitaxel, doxorubicin, or endocrine therapy, may be considered whenever appropriate (47). Therefore, treatment selection should be individualized according to prior therapy, biomarker profile, pace of disease, receptor status, performance status, and treatment goals.

#### 3.3.3 Supportive and palliative care

Supportive and palliative care should be integrated early in the management of advanced or recurrent EC rather than deferred to end-stage disease. A Cochrane review found that early palliative care improves health-related quality of life and reduces symptom intensity (48). This approach is essential, especially in patients with symptomatic burden, repeated lines of systemic therapy, functional decline, or limited expected benefit from further anticancer treatment (**Table 3**).

**Table 3:** Consensus statements regarding the systemic treatment standards.

No	Consensus statement	Agreement^ǂ^
16	Carboplatin–paclitaxel chemotherapy plus immunotherapy should be considered the standard first-line option for primary advanced or recurrent endometrial cancer.^*^	Strong consensus (100%)
17	First-line treatment in advanced or recurrent endometrial cancer should be guided primarily by molecular subtyping, including MMR immunohistochemistry (IHC), Estrogen Receptor (ER) expression, and p53 status, to determine eligibility for immune checkpoint inhibitors.^*^	Strong consensus (90%)
18	Patients with MMRd/MSI-H tumors should receive first-line carboplatin-paclitaxel in combination with immunotherapy.^*^	Strong consensus (100%)
19	Immune checkpoint inhibitors should not be routinely used as monotherapy in pMMR endometrial cancer due to their limited clinical efficacy in this subgroup.^*^	Strong consensus (100%)
20	The combination of PARP inhibitors with immunotherapy as maintenance treatment may be considered in selected fit patients with high-risk pMMR endometrial cancer, such as those with TP53 abnormalities, high-grade serous histology, or other adverse molecular and clinicopathologic features.^***^	Strong consensus (100%)
21	Endocrine therapy (progestins, AIs, tamoxifen, or fulvestrant) should be considered in ER-positive NSMP tumors, particularly in patients with indolent disease or chemotherapy intolerance.^*^	Strong consensus (100%)
22	In MMR-proficient (pMMR) and MMR-deficient (MMRd/MSI-H) tumors progressing after platinum therapy, immunotherapy-targeted combinations should be considered a standard of care in immunotherapy naïve patients.^*^	Strong consensus (100%)
23	There is currently no established standard of care for patients who experience progression following first-line therapy. However, platinum rechallenge may be considered in selected patients who previously responded to platinum-based therapy, while other systemic options, including doxorubicin, weekly paclitaxel, or endocrine therapy, could be considered.^**^	Strong consensus (100%)
24	Antibody–drug conjugates and targeted therapies should be used based on selected biomarkers as evidence evolves.^***^	Strong consensus (100%)
25	Rechallenge with immune checkpoint inhibitors in MMRd/MSI-H tumors may be considered in select patients with limited progression, durable prior response, and limited alternative treatment options.^***^	Strong consensus (100%)
26	Early supportive and palliative care should be integrated alongside systemic therapy in patients with advanced or recurrent endometrial cancer.^*^	Strong consensus (100%)

*International guideline-aligned, **GCC-specific adaptation, ***Expert-opinion recommendation.

**^ǂ^**Agreement reflects the proportion of panellists voting agree/strongly agree (scores 4–5 on a 5-point Likert scale). “Consensus” denotes 75–89% agreement, and “Strong consensus” denotes ≥90% agreement. Agreement was calculated from 12 voters, with neutral/abstain responses excluded from the denominator for the overall agreement percentage.

EC, endometrial cancer.

### 3.4 Implementation and quality improvement in advanced endometrial cancer

Effective implementation of EC care in the GCC requires coordinated referral pathways that link gynecology, oncology, pathology, and molecular diagnostics within a multidisciplinary team framework. Such pathways are essential to avoid delays in diagnosis, molecular classification, treatment planning, and reassessment at recurrence or progression (11). In addition, advanced EC management is increasingly biomarker-driven and continuously evolving; therefore, structured clinician education is essential. A nationwide survey of oncologists in the United Kingdom found that 38.7% had not received formal genomics training and 92.7% identified a need for additional training (49). Training programs should be evidence-based and regularly updated through continuing medical education programs at all levels of practice, regardless of seniority. Education programs should include molecular classification, biomarker interpretation, treatment sequencing, and multidisciplinary decision-making, rather than relying on informal or *ad hoc* learning. Data infrastructure is a further priority. Data infrastructure is a priority to support the collection of demographic, stage, histologic and molecular subtype, treatment, and outcome data, which is essential for evaluating quality of care, benchmarking practice, and identifying system-level gaps in advanced EC management.

National and regional registries should capture stage, molecular subtype, treatment patterns, and real-world outcomes to enable benchmarking, identify system-level gaps, and assess the effectiveness of biomarker-directed care in routine practice. They also acknowledged the importance of incorporating patient perspectives as guidance for quality improvement. Routine collection of patient-reported outcomes (PROs) can help monitor symptom burden, functional status, and health-related quality of life, while informing service improvement and supportive care planning (47, 50). In parallel, they highlighted that AI-integrated solutions and clinical decision support systems may help improve adherence to evidence-based pathways by integrating staging, biomarker results, and treatment eligibility into routine care workflows (**Table 4**). A systematic review by Pawloski et al., which focused on oncology practice, concluded that clinical decision support systems (CDSS) incorporated into clinical pathways were associated with increased guideline adherence across multiple studies (51). However, such tools should be implemented cautiously, validated locally, and used to support rather than replace multidisciplinary clinical decisions.

**Table 4:** Consensus statements on implementation and quality improvement in advanced endometrial cancer across GCC countries.

No	Consensus statement	Agreement^ǂ^
27	Clearly defined referral pathways between gynecology, oncology, pathology, and molecular diagnostics are essential for timely patient management, guided by a Multidisciplinary Team (MDT) approach that provides continuous oversight from initial diagnosis to potential recurrence or disease progression.^*^	Strong consensus (100%)
28	Structured education and training programs are required to improve clinician familiarity with molecularly driven treatment strategies.^*^	Strong consensus (100%)
29	National and regional registries should be established to capture real-world clinical outcomes in advanced endometrial cancer.^*^	Strong consensus (100%)
30	Patient-reported outcomes should be incorporated into routine clinical practice to inform continuous quality improvement initiatives.^*^	Strong consensus (100%)
31	AI-integrated solutions and clinical decision support systems should be implemented in GCC countries to enhance adherence to evidence-based guidelines.^*^	Strong consensus (100%)

*International guideline-aligned.

**^ǂ^**Agreement reflects the proportion of panelists voting agree/strongly agree (scores 4–5 on a 5-point Likert scale). “Consensus” denotes 75–89% agreement, and “Strong consensus” denotes ≥90% agreement. Agreement was calculated from 12 voters, with neutral/abstain responses excluded from the denominator for the overall agreement percentage.

## 4 Discussion

This is the first modified-Delphi consensus that provides a GCC-specific framework for the management of advanced and recurrent EC in an era of molecularly guided care. Persistent regional variation in diagnostics, treatment access, and care pathways were identified. In addition, the relevance of biomarker-driven treatment selection in routine practice is increasing. Available data suggest that the burden of EC is rising across the GCC, likely in parallel with the regional prevalence of obesity, diabetes, and metabolic syndrome (15). Early diagnosis, clear referral pathways, and regionally relevant evidence are required to support service planning.

The main conclusion of this consensus is that molecular classification should be embedded in routine EC care. MMR/MSI status now has direct therapeutic implications, particularly because it is the most established predictive biomarker for benefit from immune checkpoint inhibition. Other biomarkers, including p53, POLE, ER, and HER2, further refine prognostic assessment and treatment selection (11). In routine practice, immunohistochemistry may be considered a more affordable approach to implementing molecular profiling, with MMR status serving as a surrogate for MSI, p53 as a surrogate for TP53, and ER assessment guiding endocrine treatment decisions. Despite increasing adoption of molecular testing in the GCC, timely testing at diagnosis is essential, and broader implementation across the GCC will require improvement in testing capacity, turnaround time, reimbursement, MDT workflows and integration into care pathways.

The therapeutic recommendations reflected the evolving evidence in the management of EC. Chemo-immunotherapy with carboplatin-paclitaxel plus an immune checkpoint inhibitor remains the preferred first-line standard for medically fit patients with advanced or recurrent EC, with the greatest benefit observed in MMRd/MSI-H tumors (32–34). Endocrine therapy retains a role in selected ER-positive NSMP disease, particularly in patients with indolent, low-volume, or less symptomatic tumors. In addition, PARP inhibitor plus immunotherapy maintenance could be considered in selected, fit patients with biologically high-risk pMMR tumors, particularly those with p53abn features, serous histology, or other adverse clinicopathologic characteristics. ADC-based strategies could also be viewed as viable options for selected biomarker-defined populations, rather than broadly applicable treatments (34). Therefore, these therapy options could be considered after MDT discussion and should be revisited with emerging confirmatory evidence.

In later lines, treatment should remain biomarker-informed, but the introduction of chemo-immunotherapy in the first-line setting has changed the second-line landscape. As a result, there is currently no universal standard of care after progression on first-line therapy beyond selected biomarker-defined populations. Pembrolizumab-lenvatinib remains a viable option for patients with pMMR disease who are immunotherapy-naïve, while checkpoint inhibitor monotherapy remains relevant in immunotherapy-naïve MMRd/MSI-H tumors (41). Outside these settings, treatment selection should be individualized according to prior therapy, biomarker profile, pace of disease, performance status, and treatment goals.

Beyond treatment selection, this consensus emphasizes that improvement in outcomes will also depend on timely access to diagnostics and multidisciplinary care within specialized centers. Multidisciplinary tumor boards, structured clinician education, regional registries, patient-reported outcomes, and early supportive and palliative care remain necessary to support real-world translation of rapidly evolving evidence. The panel supports the development of dedicated gynecologic oncology teams within fewer referral cancer centers. Greater site specialization may represent one of the most effective strategies to improve quality of care, consistency of practice, patient experience, and overall outcomes across the GCC. Overall, this consensus provides a practical regional foundation for more consistent, biomarker-informed, and patient-centered management of advanced EC in the GCC.

## 5 Limitations

This consensus has some limitations. The modified Delphi approach depends on expert judgment and the panel’s specialties. The panel was mainly composed of medical oncologists, with limited representation from other relevant specialties and no patient representatives. The absence of radiation oncologists, palliative care specialists, oncology nurses, and patient representatives is considered a methodological limitation. In addition, real-world data on molecular subtype distribution, biomarker testing, treatment sequencing, access, and outcomes for GCC countries are limited. Access to molecular diagnostics, immunotherapy, targeted therapies, and multidisciplinary care also varies across GCC countries and between public and private healthcare settings. In addition, biomarker-guided treatment in EC is rapidly evolving, and recommendations should be interpreted in the context of evidence maturity, local access and approvals, and decisions should be individualized through multidisciplinary decision-making. Regarding the voting experts, twelve experts were included in both voting rounds and the panel meeting; however, one voting expert was not included among authors as per his preference. Finally, the consensus was supported by a targeted literature review rather than a formal systematic review. Additional databases and grey-literature sources were not searched, and a PRISMA-style flow diagram was not developed.

## 6 Conclusions

In conclusion, this consensus provides a practical, evidence-based framework to support the management of advanced and recurrent EC across the GCC. It emphasizes the need to strengthen referral pathways and multidisciplinary decision-making, standardize molecular testing, improve equitable access to biomarker-guided therapies, and expand the generation of regional real-world data. Routine molecular classification, biomarker-informed systemic treatment, and early integration of supportive care as core components of contemporary EC management are recommended. Collectively, these recommendations aim to promote more consistent, precise, and patient-centered care across GCC healthcare systems.

## Data Availability

The original contributions presented in the study are included in the article/**Supplementary Material**, further inquiries can be directed to the corresponding author.
