# Supplementary material for: Advanced endometrial cancer in the gulf cooperation council: a modified-Delphi consensus on unmet needs, molecular testing, and treatment sequencing
Source: Front Oncol. 2026 Sep 24;16:1916197. doi: 10.3389/fonc.2026.1916197 (PMC13645606; doi:10.3389/fonc.2026.1916197)
Supplement: Supplementary file 1 [file Table1.docx]

**Supplementary Methods**

**Literature search and data synthesis**

The PubMed search strategy combined Medical Subject Headings (MeSH) and free-text keywords related to advanced or recurrent endometrial cancer and the five predefined review domains using Boolean operators. The following terms were used alone and in combination: (“endometrial cancer” OR “endometrial carcinoma” OR “uterine cancer” OR “advanced endometrial cancer” OR “recurrent endometrial cancer” OR “metastatic endometrial cancer”) AND (“epidemiology” OR “incidence” OR “prevalence” OR “burden” OR “unmet need” OR “Gulf Cooperation Council” OR “GCC” OR “Middle East” OR “Bahrain” OR “Kuwait” OR “Qatar” OR “United Arab Emirates” OR “UAE” OR “Saudi Arabia” OR “Oman”) AND (“diagnosis” OR “diagnostic work-up” OR “staging” OR “FIGO 2023” OR “molecular classification” OR “molecular testing” OR “MMR” OR “MMRd” OR “MSI-H” OR “pMMR” OR “POLE” OR “p53 abnormal” OR “p53abn” OR “NSMP” OR “HER2” OR “ER” OR “biomarker”) AND (“first-line therapy” OR “first-line treatment” OR “carboplatin” OR “paclitaxel” OR “chemotherapy” OR “immunotherapy” OR “immune checkpoint inhibitor” OR “pembrolizumab” OR “dostarlimab” OR “durvalumab” OR “atezolizumab”) AND (“second-line therapy” OR “subsequent therapy” OR “treatment sequencing” OR “post-platinum” OR “lenvatinib” OR “pembrolizumab lenvatinib” OR “antibody-drug conjugate” OR “trastuzumab deruxtecan” OR “sacituzumab govitecan” OR “targeted therapy” OR “PARP inhibitor” OR “olaparib” OR “endocrine therapy”) AND (“supportive care” OR “palliative care” OR “patient-reported outcomes” OR “quality of life” OR “multidisciplinary team” OR “referral pathway” OR “clinical decision support” OR “registry” OR “implementation” OR “guideline”).
